# Cervical dystonia in a patient with cervical hydromyelic cavity

**DOI:** 10.1007/s10072-023-06984-6

**Published:** 2023-08-18

**Authors:** Andrea Quattrone, Umberto Sabatini, Maurizio Morelli

**Affiliations:** 1 Department of Medical and Surgical Sciences, Institute of Neurology, Magna Graecia University, Catanzaro, Italy; 2 Department of Medical and Surgical Sciences, Institute of Neuroradiology, Magna Graecia University, Catanzaro, Italy

**Keywords:** Cervical dystonia, Hydromyelia, Syringomyelia, Cervical spine

A 42-year-old gentleman presented with a 12-month history of neck stiffness and right head turning. He reported a minor head trauma at the age of 15 years old. The clinical picture was dominated by cervical dystonia (right torticollis) with clear gestes antagonistes, associated with dystonic posturing of the right hand while writing (video). The segmental dystonia was combined with mild cerebellar and pyramidal signs. There were no sensory symptoms/signs. Brain MRI was unremarkable, while cervical spine MRI showed a large hydromyelic cavity (see Fig. 1). The neurophysiology study showed increased blink reflex recovery cycle, as commonly reported in dystonia, [1] and increased latency of somatosensory and motor evoked potentials, likely due to a compressive effect on corticospinal and spinothalamic tracts. The clinical picture and hydromyelic cavity were unchanged at six-month follow-up. This patient was diagnosed with secondary dystonia possibly due to hydromyelic cavity. The possible underlying mechanism which has been proposed is the alteration of sensory inputs, which may lead to a disruption of the dystonia network, and the results of our neurophysiology study are in line with this hypothesis; however, a causative association has not been definitively established [2].

**Fig. 1:**
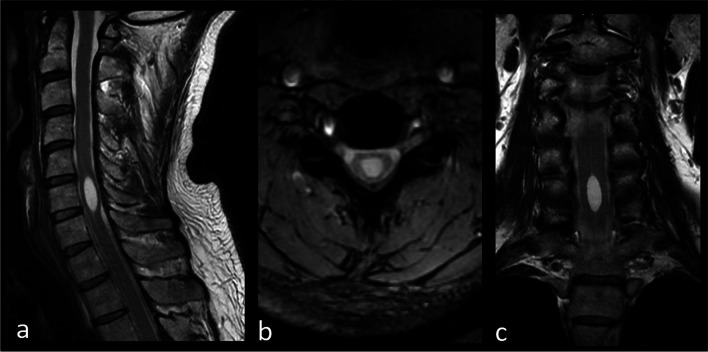
Cervical spine MRI study. T2-weighted images on sagittal (**a**), axial (**b**) and coronal (**c**) planes. The figure shows a focal fluid-filled cavity centrally located within the spinal cord at C6 level. This dilation is lined by the normal ependymal lining of the central canal and corresponds to a hydromyelic cavity

### Supplementary Information

Below is the link to the electronic supplementary material.

Supplementary file1 (MP4 15080 KB)

## Data Availability

Not applicable.
